# RecA Inhibitor Mitigates Bacterial Antibiotic Resistance †

**DOI:** 10.3390/microorganisms13092087

**Published:** 2025-09-07

**Authors:** Jin Ma, Liwen Xu, Keke Shang, Qing-Yu He, Gong Zhang

**Affiliations:** Key Laboratory of Functional Protein Research of Guangdong Higher Education Institutes and MOE Key Laboratory of Tumor Molecular Biology, Institute of Life and Health Engineering, Jinan University, Guangzhou 510632, China; majin.edu@outlook.com (J.M.); 15071052967@163.com (L.X.); shangkeke5365@126.com (K.S.); tqyhe@jnu.edu.cn (Q.-Y.H.)

**Keywords:** bacterial resistance, *Escherichia coli*, translation, RecA inhibitor, RecA, tRNA

## Abstract

Bacterial antibiotic resistance (AR) has become a critical global health threat. AR is mainly driven by adaptive resistance mutations and the horizontal gene transfer of resistance genes, both of which are enhanced by genome recombination. We previously discovered that genome recombination-mediated tRNA upregulation is important for AR, especially in the early stages. RecA is a crucial bacterial factor mediating genome recombination and the DNA damage response. Therefore, RecA inhibitors should be effective in reducing AR. In this study, we found that BRITE-338733 (BR), a RecA inhibitor, can prevent ciprofloxacin (CIP) resistance in subculturing *Escherichia coli* strain BW25113 in the early stages (up to the 7th generation). In the presence of BR, the tRNA was decreased, so the bacteria cannot evolve resistance via the tRNA upregulation-mediated AR mechanism. The RecA expression level was also not increased when treated with BR. Transcriptome sequencing revealed that BR could inhibit oxidative phosphorylation, the electron transport chain process, and translation, thereby reducing the bacterial energy state and protein synthesis. Also, the effective concentrations of BR do not harm human cell viability, indicating its clinical safety. These findings demonstrate that BR effectively delays the emergence of spontaneous AR by targeting RecA-mediated pathways. Our findings shed light on a new strategy to counteract clinical AR: applying BR with the antibiotics together at the beginning.

## 1. Introduction

Since the introduction of penicillin in the 1940s, antibiotics have significantly reduced the burden of bacterial infections, including pneumonia, sepsis, and wound infections [1]. However, the widespread and often inappropriate use of antibiotics has accelerated the emergence and dissemination of antibiotic-resistant bacteria. This trend poses a serious threat to global public health and clinical treatment effectiveness. According to a comprehensive analysis, antimicrobial resistance (AR) was directly responsible for an estimated 1.27 million deaths in 2019 and contributed to nearly 5 million deaths globally. Without effective intervention, AMR-related deaths are projected to rise to 10 million annually by 2050, with cumulative deaths exceeding 39 million from 2025 to 2050 [2].

The development of resistance is not limited to acquired resistance through horizontal gene transfer, such as plasmids or transposons [3]. Accumulating evidence indicates that many bacterial strains rapidly develop resistance to antibiotics, including newly introduced synthetic antibiotics, shortly after their clinical application. These cases cannot be explained solely by traditional acquired resistance theories, highlighting the critical role of spontaneous (or intrinsic) resistance mechanisms [4,5]. Spontaneous resistance involves innate genomic adaptations such as mutations in drug targets, changes in membrane permeability, activation of efflux pumps, and reprogramming of gene expression, including tRNA rearrangements, without the involvement of exogenous genes [6,7].

Our previous studies have shown that under antibiotic-induced oxidative stress, bacteria can rapidly upregulate tRNA levels through RecA-mediated genome recombination, sustaining translational elongation and survival [8,9]. This early-stage adaptation provides bacteria with a generalized resistance strategy, even in the absence of specific resistance genes or target mutations [8]. Therefore, early intervention against this RecA-mediated adaptive process may delay or prevent the emergence of drug-resistant strains.

RecA plays a central role in bacterial homologous recombination and SOS response in almost all bacteria [10]. We and other researchers have found that RecA deletion reduces the resistance of clinical *E. coli* strains against multiple antibiotics [11]. Inhibition of RecA activity can disrupt bacterial DNA repair and reduces the mutagenesis of *E. coli* that was exposed to antibiotics [12]. In contrast, elevated RecA expression enhances the AR of *Proteus* spp. strains against ciprofloxacin [13]. These results indicated that the RecA should be a universal target of AR in bacteria. Although knocking out RecA is not possible in clinical treatment, we postulate that inhibiting RecA using RecA inhibitors may serve as a practical way to prevent or delay AR evolution. Several chemotypes of RecA inhibitors have been described with two main modes of action: (i) compounds that block ssDNA-stimulated ATP hydrolysis and/or destabilize RecA–ssDNA filaments, thereby preventing recombination and the co-protease activity that drives SOS induction; and (ii) agents that interfere with filament assembly on ssDNA and reduce recombination-dependent mutagenesis [14].

High-throughput screening has identified the compound BRITE-338733 (BR), a 2-amino-4,6-diarylpyridine derivative with potent RecA ATPase inhibition (${\mathrm{IC}}_{50}$ = 4.7 μM) [15]. In this study, we constructed a long-term adaptation model using *E. coli* BW25113 exposed to sublethal doses of various antibiotics, with or without BR co-treatment. We evaluated the effect of BR on bacterial growth kinetics, tRNA regulation, RecA expression, and transcriptome dynamics, aiming to elucidate the molecular mechanism by which BR suppresses spontaneous AR [16]. This study provides a potential pharmacological strategy for inhibiting early resistance mechanisms and preventing the escalation to high-level resistance.

## 2. Materials and Methods

### 2.1. Bacterial Strains and Cell Lines

*Escherichia coli* strain BW25113 was purchased from the Coli Genetic Stock Center of Yale University (New Haven, CT, USA) [17]. The reference genome sequence NZ_CP009273.1 and its annotation (downloaded from NCBI) were used in the bioinformatics. The human normal lung epithelial cell line BEAS-2B and human non-small cell lung cancer cell lines A549, H292, and H1299 used in this study were obtained from our research group and purchased from ATCC [18]. During cell culture, strict mycoplasma testing was performed regularly to ensure that the cells were free of mycoplasma contamination.

### 2.2. Serial Passage of Bacteria with Antibiotics and RecA Inhibitor

The RecA inhibitor used in this study was BRITE-338733 (2-(4-(5-ethylfuran-2-yl)-6-(2,2,6,6-tetramethylpiperidin-4-ylamino)pyridin-2-yl)-4-methylphenol), purchased from Shanghai Yuanye Bio-Technology Co., Ltd. (Shanghai, China). CAS: 503105-88-2; formula $C_{27}$$H_{35}$$N_{3}$$O_{2}$; molecular weight (MW) 433.5857; purity ≥98%. BR is a synthetic small molecule originally sourced from the BRITE diversity compound library (Biogen-Idec, Cambridge, MA, USA) [15]. For experiments, a concentrated stock was prepared in 95% ethanol, aliquoted, protected from light, and stored at −20 °C; working solutions (0.1–10 μM) were freshly prepared in culture medium. The final vehicle (ethanol) content was kept at ≤0.1% (*v*/*v*) in all conditions, and vehicle controls were included. Identity of the batch used was confirmed ${\mathrm{by}\;}^{1}$H NMR (Figure A2).

In this study, the inhibitory effect of BR on the initial universal resistance mechanisms of bacteria during adaptation to antibiotics was tested using the sensitive strain BW25113 [15]. The experiment consisted of two groups: the single antibiotic adaptation group (control) and the BR-antibiotic combination adaptation group.

For the single antibiotic adaptation group, BW25113 was cultured in LB medium from a single colony as generation 0. Bacteria were inoculated at a 1:500 dilution into fresh LB medium containing 1/2 minimum inhibitory concentration (MIC) antibiotics and incubated at 37 °C with shaking at 200 rpm for 12 h [19]. MICs were re-evaluated every 12 h, and this process was repeated for 15 generations.

For the BR-antibiotic combination adaptation group, six concentrations of BR (10 μM, 5 μM, 1 μM, 0.5 μM, 0.2 μM, and 0.1 μM) were tested in combination with five antibiotics: ciprofloxacin (CIP), ampicillin (AMP), polymyxin B (PMB), kanamycin (KAN), and tetracycline (TET). BR was used to inhibit the bacteria’s initial universal resistance mechanisms, such as tRNA rearrangement, during adaptation. Bacteria were subjected to the same 12 h cycle, and MICs were determined for each generation to evaluate the inhibitory effect of BR on resistance development.

MICs were measured for each generation using 48-well plates containing serial dilutions of antibiotics, and the lowest concentration with OD600 < 0.1 was recorded as the MIC [20]. Bacterial populations from each generation were pooled and subjected to transcriptomic sequencing and bioinformatics analysis to characterize the molecular dynamics underlying adaptive evolution and antibiotic resistance development.

### 2.3. Assessment of Mammalian Cell Proliferation and Viability

Cells in the logarithmic growth phase were seeded into a 96-well plate at a density of 5000 cells per well, with three replicates for each drug concentration. A drug gradient was prepared by adding drugs to the culture medium at a volume of 200 μL per well to achieve the following final concentrations: 0.06, 0.12, 0.24, 0.49, 0.98, 1.95, 3.91, 7.81, 15.63, 31.25, 62.50, and 125 μM. After the cells adhered to the plate, the supernatant was removed, and 200 μL of the drug-containing culture medium was added to each well. The cells were incubated for 48 h in a humidified incubator. Following the incubation, CCK-8 solution was added to each well, and the absorbance at 450 nm was measured. The absorbance values were plotted against the drug concentrations to analyze the effects of different drug concentrations on cell proliferation.

### 2.4. Quantitative Analysis of tRNA Expression by Gel Electrophoresis

Equal amounts of RNA samples from a selected generation were loaded and subjected to electrophoresis. The agarose electrophoresis was performed at 120 mV for 30 min on a 1% agarose gel, and the polyacrylamide electrophoresis was run at 120 mV for 120 min on a 15% polyacrylamide gel. The grayscale of the rRNA and tRNA bands was measured and analyzed using Adobe Photoshop (version 13.0).

### 2.5. Transcriptome Sequencing

The bacterial cells were harvested by centrifugation at 4 °C, 4000× *g* for 5 min. The cell pellet was resuspended and washed using PBS. The cells were treated with using 1.25 mg/mL lysozyme at 4 °C for 10 min and collected by centrifugation at 5000× *g* for 5 min. The pellet was dissolved in 1 mL Trizol reagent, and the RNA was extracted using the Trizol method. Total RNA was extracted from bacterial samples collected from two repeated experiments for each group. RNA sequencing was performed by Chi Biotech Co., Ltd. (Shenzhen, China) and Novogene Co., Ltd. (Beijing, China). After library construction and sequencing, mapping and analysis were performed by Chi-Cloud with FANSe3 [21].

Gene expression levels were quantified as read counts and RPKM (reads per kilobase per million mapped reads). Pearson correlation coefficients (R) were calculated between samples and visualized using the pheatmap package in R software (version 3.5.3). Hierarchical clustering and correlation heatmaps were generated to evaluate data consistency [22,23].

Differentially expressed genes (DEGs) were identified using the DESeq2 package (version 1.26.0), with thresholds of fold change >2 and adjusted *p*-value < 0.05 [24]. GO and KEGG enrichment analyses were performed using the clusterProfiler R package (version 3.14.3) [25]. GO terms were categorized into biological process (BP), molecular function (MF), and cellular component (CC). Enriched pathways related to translation, ribosome structure, electron transport, and oxidative metabolism were analyzed to understand transcriptional changes under antibiotic stress and BR treatment.

### 2.6. Bacterial Growth Curve Analysis

Bacterial growth (OD600) was monitored using a growth detection device (Chi Biotech Co., Ltd., Zhang’s Laboratory). The bacteria were incubated at 37 °C with shaking for 12 h. Data before 2 h were excluded to account for the adaptation phase, and data after 2 h were used for curve fitting.

The bacterial growth data were analyzed using the corrected Gompertz model (Equation (1)) and fitted with Origin 2021 software (OriginLab, Northampton, MA, USA) [26].

(1) $$\mathrm{lg}\left(\frac{N_{t}}{N_{\min}}\right)=\mathrm{lg}\left(\frac{N_{\max}}{N_{\min}}\right)\times \exp\left\{-\exp\left[\mu_{\max}\times 2.718\times \frac{\lambda -t}{\mathrm{lg}\left(\frac{N_{\max}}{N_{\min}}\right)}+1\right]\right\}$$

where *t* represents time (min); $N_{t}$ is the absorbance at time *t*; $N_{\max}$ and $N_{\min}$ are the maximum and minimum OD values, respectively; $\mu_{\max}$ is the maximum growth rate ($\min^{-1}$); and λ is the lag phase duration (min).

### 2.7. Western Blotting

The bacterial protein was isolated after bacterial lysis and was quantified by Pierce Quantitative Colorimetric Peptide Assay (Thermo, Waltham, MA, USA). After being separated by SDS-PAGE, the protein was transferred to a PVDF membrane and blocked with 5% milk for 2 h. The membrane was incubated with the primary antibody at 4 °C overnight, followed with incubation of the corresponding secondary antibody at room temperature for 1 h. The protein signal bands were detected and visualized by enhanced chemiluminescence.

### 2.8. Statistical Analysis

All data in this study were analyzed and plotted using GraphPad Prism 10.1.2 for Windows (GraphPad Prism Software, San Diego, CA, USA). One-way analysis of variance (ANOVA) was used to analyze the mean difference in the normal distribution data. For data that did not follow a normal distribution, the Kruskal–Wallis (KW) test was applied to assess group differences. * p≤0.05, **p≤0.01, ***p≤0.001, or ****p≤0.0001; these experimental results were considered to have significant differences.

## 3. Results

### 3.1. BR Suppresses CIP-Induced Resistance Development in E. coli BW25113

**Figure 1:**
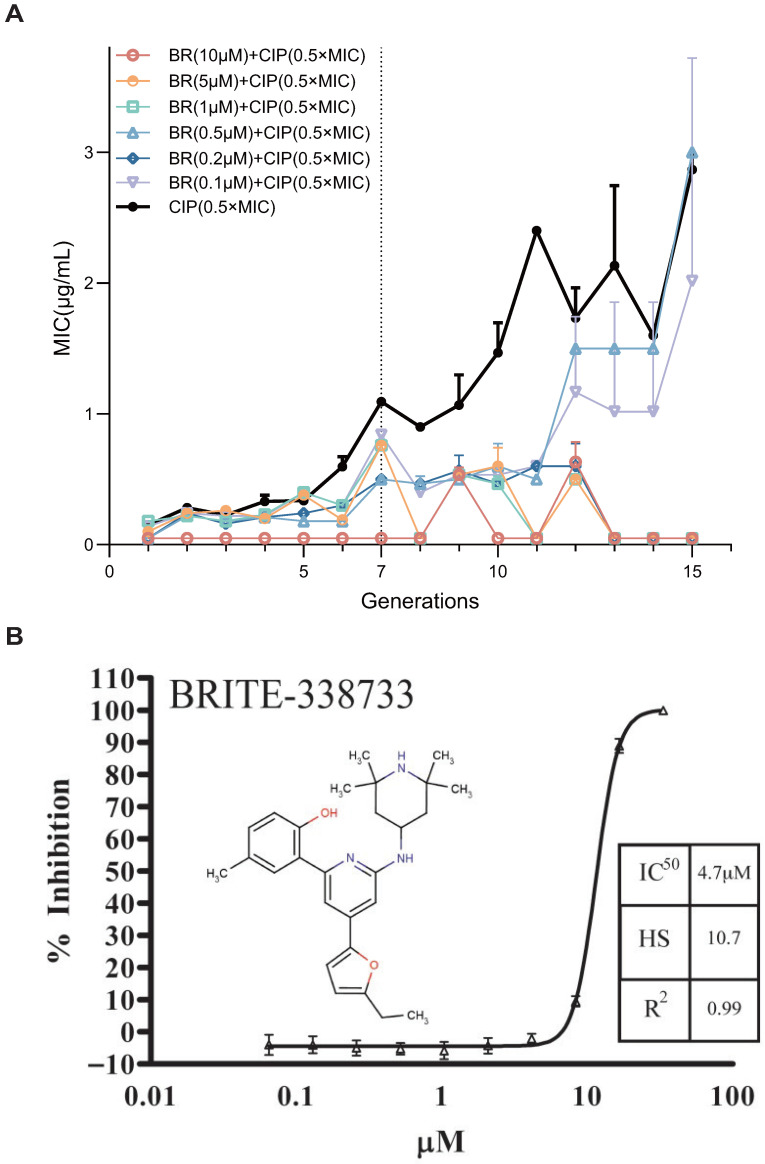
Biochemical potency of BRITE-338733 (BR) against RecA and its effect on ciprofloxacin resistance evolution in *Escherichia coli* BW25113. (**A**) Inhibitory effect of different concentrations of BR combined with sublethal CIP (0.5× MIC) on resistance development in BW25113. (**B**) Dose-response curve for the RecA inhibitor BRITE-338733, exhibiting an ${\mathrm{IC}}_{50}$ value of 4.7 ± 0.5 μM and characteristic steep Hill-slope [15].

To evaluate the ability of BR, a RecA inhibitor, to suppress the spontaneous development of AR, we established an in vitro resistance evolution model. BR’s chemical structure and its RecA ATPase dose-response (${\mathrm{IC}}_{50}$ = 4.7 μM) are shown in Figure 1B [15]. *Escherichia coli* BW25113 was serially passaged for 15 generations under sub-inhibitory pressure of CIP (0.5× MIC), with or without six concentrations of BR (10, 5, 1, 0.5, 0.2, and 0.1 μM). MIC values were recorded every generation to evaluate the kinetics of resistance acquisition (Figure 1A) [27].

In the absence of BR, the MIC of CIP increased steadily with generations, indicating successful resistance evolution. BR at high concentrations (10, 5, 1, and 0.2 μM) completely suppressed MIC elevation throughout all 15 generations. BR at 0.5 μM and 0.2 μM showed partial inhibition, delaying MIC increase until later generations (Generation 12–15).

These findings suggest that BR can dose-dependently suppress early-stage resistance adaptation to CIP. Notably, BR at concentrations ≥1 μM can fully inhibit resistance development, likely by interfering with the key molecular pathways required for early-stage adaptation.

### 3.2. BR Selectively Inhibits Antibiotic Resistance Development Without Significant Cytotoxicity in Mammalian Cells

To assess the safety profile of BR within its effective concentration range, we evaluated its effects on both bacterial and human cell proliferation. Four representative human cell lines—H1299, A549, BEAS-2B, and H292—were exposed to BR at concentrations ranging from 0.06 μM to 125 μM for 48 h. Cell viability was assessed using the CCK-8 assay.

**Figure 2:**
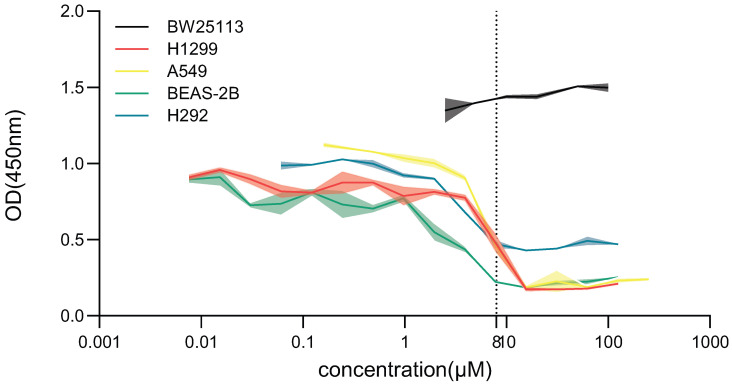
Selective effects of BR on bacterial and human cell proliferation. BW25113 and four human cell lines were treated with BR (0.001–125 μM) for 48 h, and OD450 was measured. BR showed no inhibition of bacterial growth within 1–10 μM and minimal cytotoxicity to human cells, indicating favorable selectivity and safety.

At BR concentrations ≥ 7.81 μM, proliferation of H1299, A549, and H292 cells was significantly suppressed, whereas BEAS-2B cells showed reduced viability only at ≥3.91 μM. In contrast, no significant cytotoxicity was observed in the 0.1–1.0 μM range across all four cell lines, indicating that BR is well tolerated at concentrations effective for resistance inhibition (Figure 2).

Furthermore, BR did not impair the growth of *E. coli* BW25113 at any concentration tested 0–100 μM, as OD450 values remained stable, confirming that BR is non-toxic to bacteria under normal culture conditions and does not affect bacterial viability in the absence of antibiotic pressure (Figure 2).

These findings confirm that BR exerts favorable selectivity, inhibiting resistance evolution while sparing bacterial growth and mammalian cell viability at therapeutic concentrations. This safety profile supports the potential clinical application of BR as an adjuvant compound in antimicrobial therapy.

### 3.3. BR Inhibits Growth Fitness and Adaptive Evolution of Drug-Resistant BW25113

To assess how BR affects bacterial growth dynamics during resistance adaptation, we monitored the growth kinetics of BW25113 strains passaged under CIP (0.5× MIC), with or without BR treatment. Bacterial growth was recorded over time and fitted to the modified Gompertz model to derive key parameters including maximum growth rate ($\mu_{\max}$) and lag time (λ).

**Figure 3:**
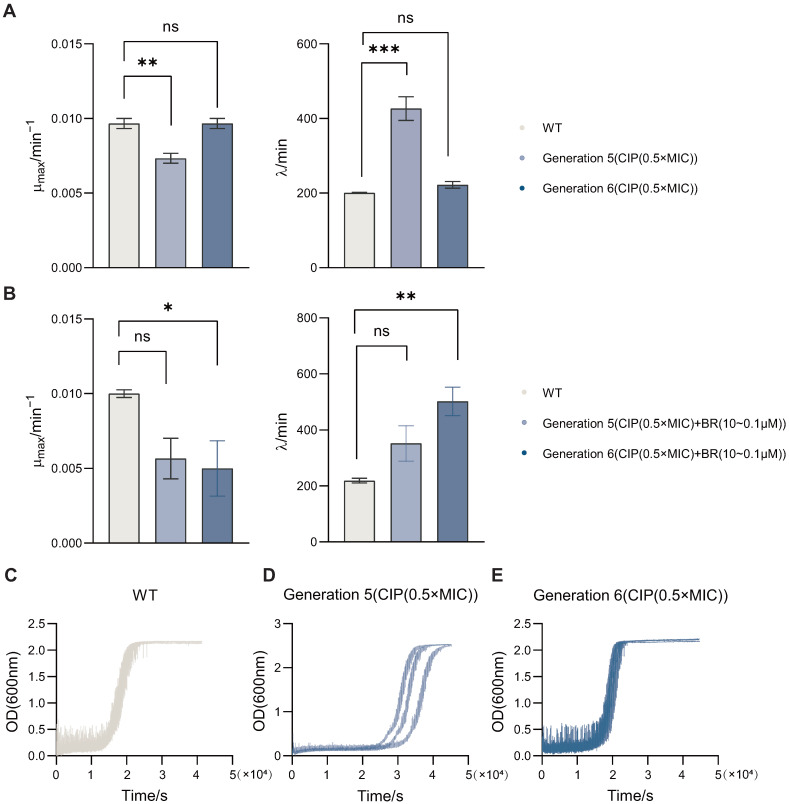
Effects of BR on BW25113 growth during CIP-induced early adaptation (Gen5–Gen6). Growth curves were fitted to the modified Gompertz model to obtain ($\mu_{\max}$) and lag time (λ). (**A**) WT and CIP-passaged strains at Generation 5 and Generation 6. (**B**) WT and CIP + BR-passaged strains at Generation 5 and Generation 6. (**C**) Representative growth curve of WT. (**D**) Representative growth curves of CIP-passaged Generation 5. (**E**) Representative growth curves of CIP-passaged Generation 6. CIP-passaged strains showed reduced $\mu_{\max}$ and prolonged λ relative to WT; BR co-treatment further decreased $\mu_{\max}$ and extended λ. “*”, *p* < 0.05; “**”, *p* < 0.01; “***”, *p* < 0.001; “ns”, not significant.

By comparing wild-type (WT) and passaged strains, we found that (Figure 3A) CIP-only passaging for five generations significantly reduced $\mu_{\max}$ (p<0.01) and prolonged λ (p<0.001) versus WT. As shown in Figure 3B, co-treatment with CIP and BR in sixth-generation strains also decreased $\mu_{\max}$ and increased λ relative to WT. Representative growth curves for WT and CIP-passaged strains (Gen5 and Gen6) are shown in Figure 3C–E. These results indicate that BR is associated with reduced $\mu_{\max}$ and extended λ during the early stage of adaptation.

Since a high $\mu_{\max}$ and short lag time are commonly associated with strong bacterial viability and potential virulence, these shifts suggest that BR may attenuate the fitness of adapting populations. Compared with CIP monotherapy, the CIP + BR combination produced larger changes—particularly at Generation 6, supporting a role for BR in suppressing early-stage spontaneous resistance development.

### 3.4. BR Maintains RecA Protein Expression and Suppresses Resistance Driven by DNA Repair

To further explore the molecular basis of BR’s inhibition of spontaneous resistance, we investigated its effect on RecA protein levels during resistance adaptation. RecA is a central regulator of homologous recombination and DNA damage repair, and its upregulation has been associated with the emergence of spontaneous AR.

**Figure 4:**
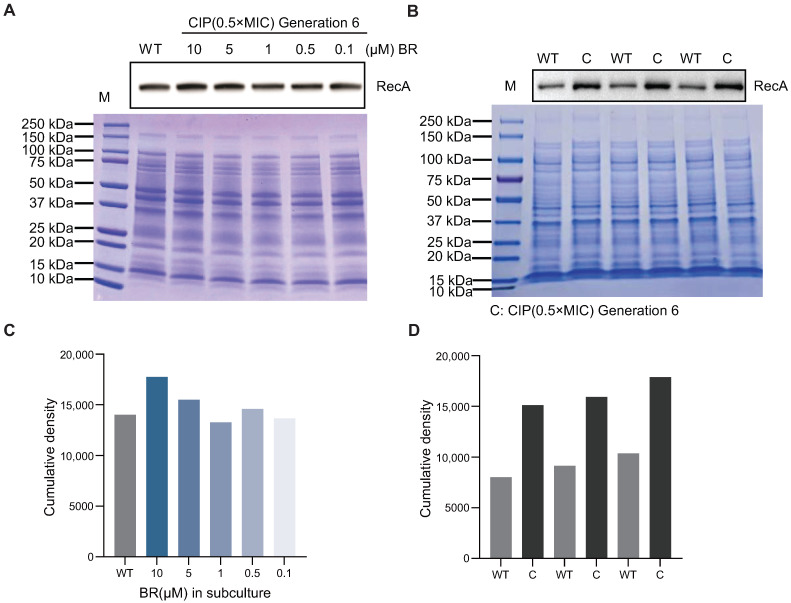
Effect of BR on RecA expression in BW25113 Generation 6 under CIP stress. (**A**) Western blot analysis of RecA in Generation 6 BW25113 treated with CIP (0.5× MIC) plus BR at 10, 5, 1, 0.5, and 0.1 μM; Coomassie-stained gel confirmed equal protein loading. BR co-treatment maintained RecA levels comparable to wild-type. (**B**) Western blot comparison of RecA between wild-type (WT) and CIP-passaged Generation 6 strains, showing that CIP alone induces RecA upregulation. (**C**,**D**) Semi-quantitative densitometry of RecA for panels A and B.

Western blotting was performed to assess RecA expression in sixth-generation BW25113 strains passaged under CIP (0.5× MIC), with or without BR co-treatment (Figure 4B,D). However, in strains co-treated with BR at 10, 5, 1, 0.5, and 0.1 μM, RecA protein levels remained comparable to wild-type levels (Figure 4A,C), showing no upregulation during the passage process [10].

In summary, BR prevents CIP-induced overexpression of RecA during early-stage adaptation, thereby suppressing DNA repair activity and limiting recombination events that may lead to tRNA amplification and the emergence of broad-spectrum resistance mechanisms.

### 3.5. BR Downregulates tRNA Expression and Interrupts the Resistance Pathway

**Figure 5:**
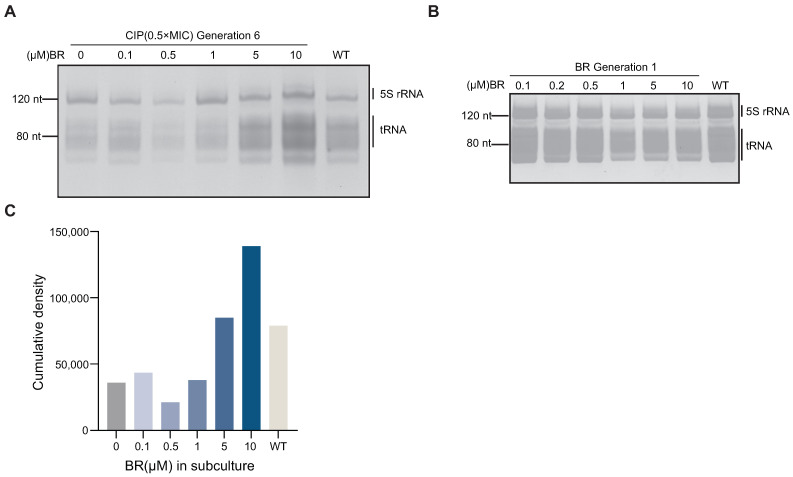
Effect of BRITE-338733 (BR) on tRNA levels in *E. coli* BW25113 during CIP adaptation. (**A**) Denaturing PAGE of 5S rRNA and tRNA extracted from Generation 6 strains treated with CIP (0.5× MIC) plus BR at 10, 5, 1, 0.5, or 0.1 μM; wild-type (WT) as reference. (**B**) Denaturing PAGE of 5S rRNA and tRNA from Generation 1 strains treated with BR alone (10, 5, 1, 0.5, or 0.1 μM); WT as control. (**C**) Semi-quantitative analysis of tRNA band intensities in Generation 6 under CIP ± BR. Co-treatment with BR markedly reduced tRNA levels in adapted cells, whereas BR alone had no effect on bacterial tRNA levels.

To investigate whether BR inhibits spontaneous resistance by suppressing RecA-mediated recombination and thereby limiting tRNA amplification, we analyzed tRNA levels in BW25113 during CIP adaptation. Total RNA was extracted from sixth-generation strains treated with CIP (0.5× MIC) combined with different concentrations of BR (10, 5, 1, 0.5, or 0.1 μM). Denaturing PAGE analysis showed that tRNA levels in BR + CIP-treated strains were significantly reduced compared to the wild-type (Figure 5A). Denaturing PAGE and fluorescence-based quantification (Figure 5A,C) revealed that tRNA levels were reduced in early-stage adapted strains (Generation 6) treated with a combination of CIP and BR (1, 0.5, and 0.1 μM), compared to the wild-type (Figure 5C).

This reduction may reflect a bacterial survival strategy of lowering translation and energy metabolism to cope with antibiotic-induced stress. The result is consistent with earlier findings that BR delays growth and reduces metabolic activity. To verify whether this effect is stress-induced, we analyzed tRNA levels in Generation 1 strains treated with BR alone (10–0.1 μM). No significant change was observed compared to the wild-type (Figure 5B), indicating that BR alone does not affect tRNA expression under normal conditions.

These findings support the conclusion that BR suppresses RecA-driven tRNA amplification and interferes with the translational machinery required for spontaneous resistance development [8].

### 3.6. BR Inhibits the Transcriptome and Suppresses Key Pathways in Resistant Strains

**Figure 6:**
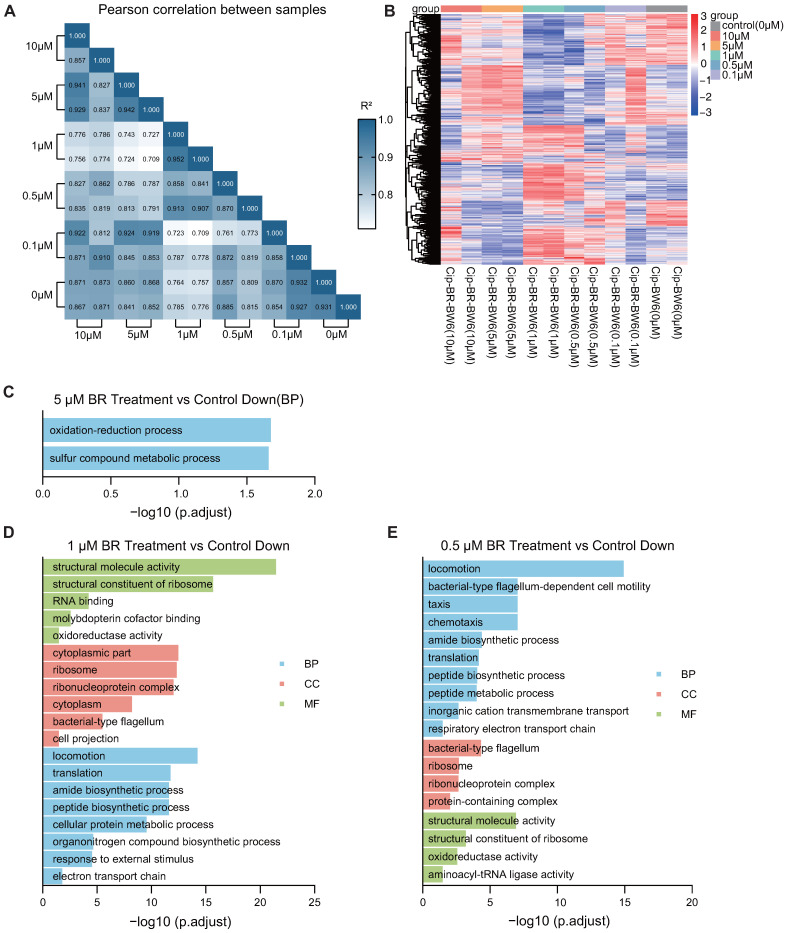
Transcriptomic reprogramming by BR during early ciprofloxacin adaptation. (**A**,**B**), RNA-seq similarity (Pearson $R^{2}$) and hierarchical clustering of Generation 6 cultures. (**C**–**E**), GO terms enriched among downregulated genes (FDR < 0.05) versus control: 5 μM—redox/sulfur metabolism; 1 μM—translation and motility; 0.5 μM—respiratory chain and chemotaxis. BR at 0.5–5 μM thus rewires energy and protein-synthesis pathways early in resistance evolution.

To explore how BR impairs the early adaptation of CIP-resistant *E. coli*, RNA-seq was performed on Generation 6 strains treated with different concentrations of BR (0.1–10 μM) in combination with CIP. The differential gene expression heatmap and correlation heatmaps showed that 1–5 μM BR treatment caused substantial shifts in the global transcriptional profiles compared to CIP-only controls, while 0.1–0.5 μM BR had weaker effects (Figure 6A,B).

Gene ontology (GO) enrichment analysis of downregulated genes revealed that BR disrupted several critical biological pathways associated with energy metabolism and translation. Specifically, 5 μM BR significantly suppressed the oxidation-reduction process and sulfur compound metabolism (Figure 6C) [28,29]; 1 μM BR led to downregulation of genes involved in translation, motility, electron transport, and protein biosynthesis (Figure 6D); and 0.5 μM BR impaired respiratory chain activity, chemotaxis, and ribosomal structure and function (Figure 6E).

**Figure A1:**
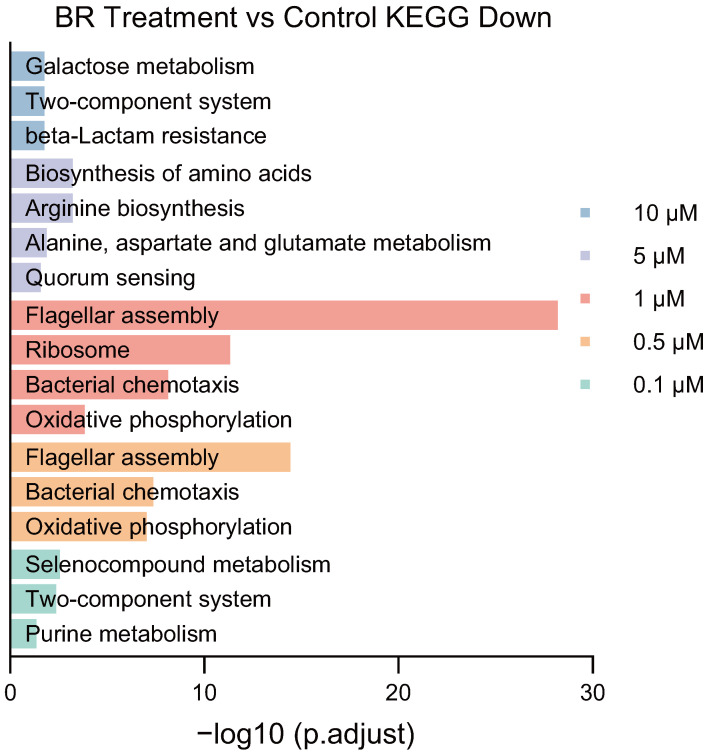
BR (0.1–10 μM) suppressed pathways related to translation, ribosome, oxidative phosphorylation, and motility, indicating impaired protein synthesis and energy metabolism during resistance adaptation.

**Figure A2:**
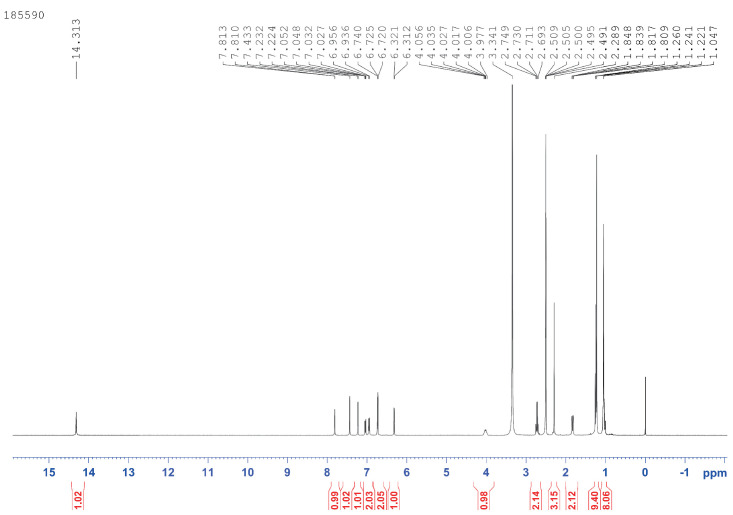
BR was purchased from Shanghai Yuanye Bio-Technology (CAS:503105-88-2; purity ≥ 98% per CoA). Shown is ${\mathrm{the}\;}^{1}$H NMR spectrum of BR used in this study. The spectrum displays the expected aromatic/heteroaromatic region, a downfield phenolic/O–H resonance, and aliphatic resonances from the tetramethylpiperidinyl and ethyl substituents, confirming compound identity.

In addition to GO enrichment, KEGG analysis further confirmed that BR treatment disrupted key signaling and metabolic pathways during early resistance adaptation. Specifically, 1 μM BR suppressed ribosome function, translation, amide biosynthetic process, peptide biosynthetic process, and cellular protein metabolic process, indicating impaired protein synthesis and energy production (Figure A1). Moreover, locomotion, flagellar assembly, and chemotaxis were significantly downregulated, suggesting a reduction in motility and nutrient foraging. Downregulation of the respiratory electron transport chain implied impaired oxidative phosphorylation and ATP production. These impairments collectively contribute to reduced bacterial viability and stress response capacity [30].

At 0.5 μM BR, KEGG results showed suppression of oxidative phosphorylation, chemotaxis, and ribosomal structural constituents. These data suggest that BR interferes with both energy metabolism and translational machinery in a concentration-dependent manner. Importantly, the suppression of ribosome biogenesis and motility-related genes may explain the bacteriostatic effect of BR during early adaptation and its inhibition of stress-induced tRNA upregulation.

These results suggest that BR concentrations ranging from 0.5 to 5 μM impair cellular energy supply and translational capacity, thereby limiting the growth and resistance adaptation of bacteria.

### 3.7. BR Exhibits Broad-Spectrum Inhibitory Effects on Spontaneous Resistance Evolution Against Multiple Classes of Antibiotics

To evaluate whether the inhibitory effect of BR on spontaneous resistance evolution is generalizable beyond CIP, we tested BR in combination with four additional antibiotics—PMB, AMP, KAN, and TET—at sublethal concentrations (0.5× MIC). BW25113 was serially passaged for 15 generations with each antibiotic alone or in combination with BR (0.1–10 μM), and MICs were determined at each generation.

**Figure 7:**
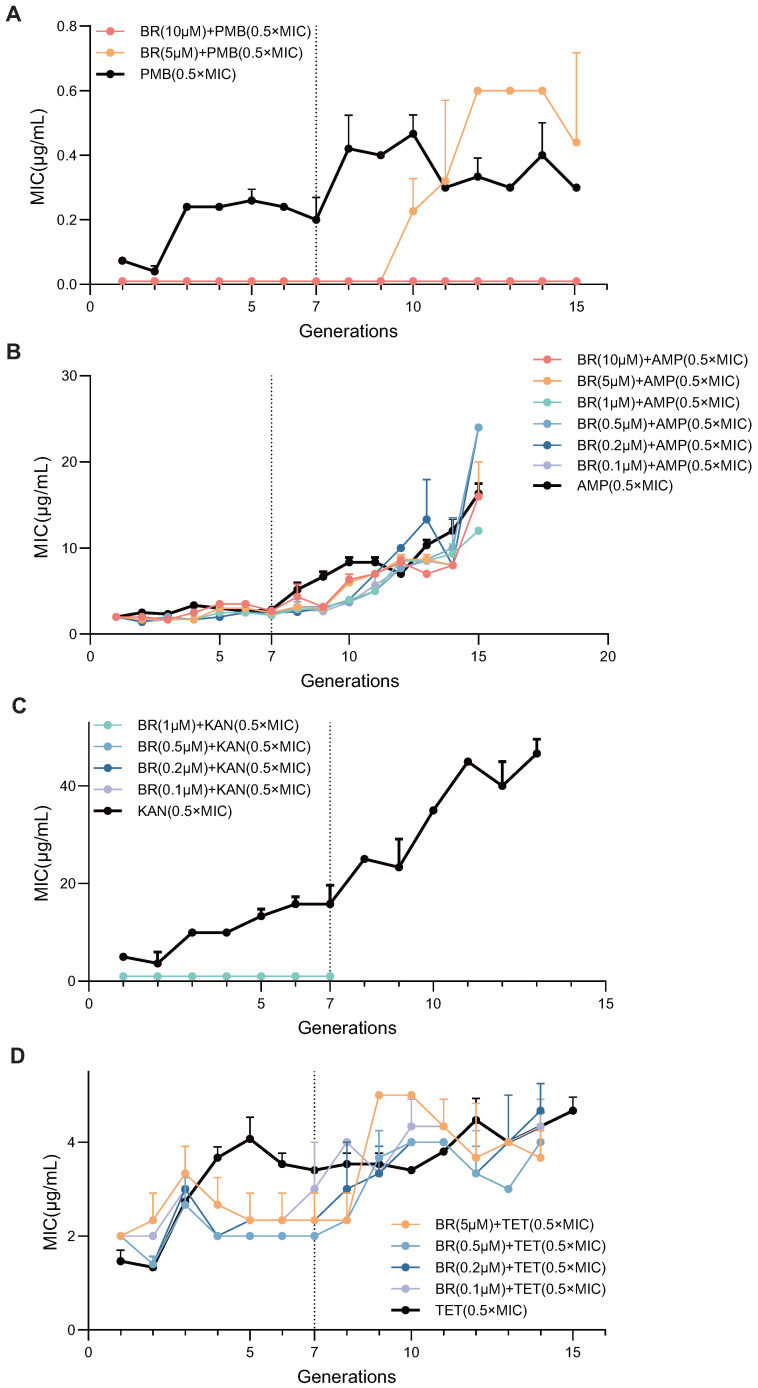
BR broadly suppresses resistance evolution in BW25113. Populations were serially passaged for 15 generations under 0.5× MIC PMB, AMP, KAN, or TET with antibiotic alone or combined with 0.1–10 μM BR. MICs were determined at each generation (mean ± SD, n=3); the dashed line marks Generation 7. (**A**–**D**) correspond to PMB, AMP, KAN, and TET. BR at ≥1 μM suppressed or blocked MIC increases to varying extents across all four antibiotics.

As shown in Figure 7, BR exerted varying degrees of suppression against resistance development depending on the antibiotic and BR concentration. For PMB (Figure 7A), 10 μM BR completely blocked MIC elevation throughout 15 generations, 5 μM BR delayed resistance up to generations 9–15. For AMP (Figure 7B), all six BR concentrations (0.1–10 μM) inhibited MIC elevation within the first nine generations; MIC increases appeared beyond Generation 9. For KAN (Figure 7C), four BR concentrations (1.0, 0.5, 0.2, and 0.1 μM) fully suppressed MIC elevation within the first seven generations. For TET (Figure 7D), BR at 5, 0.5, 0.2, and 0.1 μM partially delayed resistance development, with MIC increases resuming beyond Generation 7.

These findings demonstrate that BR can attenuate early resistance adaptation across diverse antibiotic classes. Although the inhibitory effectiveness varied depending on BR dose and antibiotic type, low concentrations of BR (≤1 μM) showed reproducible suppressive effects against resistance emergence under KAN and TET pressure. This suggests the potential applicability of BR as a general adjuvant to suppress the emergence of AR.

## 4. Discussion

AR poses a critical challenge to global health, often rendering conventional treatments ineffective. Traditionally, antibiotic resistance has primarily been attributed to the horizontal acquisition of resistance determinants such as plasmids, integrons, or transposons from environmental reservoirs [1]. Yet resistance emerges soon after the clinical introduction of entirely synthetic antibiotics, which cannot be explained by gene acquisition. This highlights the importance of spontaneous (intrinsic) resistance—adaptation without foreign DNA—driven by target mutations, altered permeability, efflux, and reprogrammed gene expression, including tRNA rearrangements [4,5,6].

Antibiotic stress induces oxidative damage that demands rapid cellular responses; while canonical regulators (*SoxRS*, *OxyR*) require ∼20–30 min to activate, translational responses can act faster [30]. In our study, BR co-treatment downregulated translation-related genes, ribosomal components, and aminoacyl-tRNA synthetases, indicating suppressed initiation of protein synthesis and blunted stress-response activation. Consistent with this, growth-kinetics analyses showed reduced $\mu_{\max}$ and prolonged λ during the early adaptation window under sub-MIC pressure, supporting a model in which BR constrains early translational and energetic capacity and limits survival during initial antibiotic insult.

The mechanism of BR brings a series of advantages and limitations, especially in the context of clinical treatment. Mechanistically, BR abolished the RecA upregulation induced by various antibiotics and thus delayed the genome recombination-mediated tRNA upregulation and the subsequent translation-based spontaneous AR at early stage. Together with the transcriptomic signatures pointing to reduced oxidative phosphorylation, electron transport, and translation, the data converge on a lower-energy, lower-protein-synthesis state under BR. Due to the highly conserved RecA and SOS repair system across bacterial species, BR should be effective in various bacteria and for most antibiotics. Since RecA-mediated AR is only for the early stage, the potential usage of BR should be accompanied with antibiotics in the very beginning of clinical treatment. Also, due to this mechanism, BR would not reverse the AR if specific AR through efflux pumps or mutations has already occurred. Therefore, BR should be used only with the antibiotics that the bacteria is sensitive to. In terms of stability, it seems that BR was stable throughout the entire process of multiple-day subculturing experiments. However, the rate of clearance in vivo should be further investigated to guide clinical practice.

Across antibiotic classes—PMB, AMP, KAN, and TET—BR attenuated early-stage adaptation to varying degrees, likely reflecting differences in drug mechanisms and induced stress pathways. Notably, suppression did not scale monotonically with dose, suggesting a nonlinear dose-response; in some settings, higher BR did not further improve control, and resistance could re-emerge via energy-conservation strategies such as growth arrest or reduced motility. This means that the dosage of BR should be investigated in depth for each bacteria and for each antibiotics. We also note a plausible link between BR exposure and persister-like, low-energy cells; second messengers (e.g., c-di-GMP) and toxin proteins such as HipH may modulate persistence and genome stability, meriting future co-targeting studies [31]. Optimizing BR-based combinations and scheduling by antibiotic class/mechanism may therefore maximize efficacy.

From a translational perspective, BR showed minimal cytotoxicity in human cell lines within the antibacterial-adjuvant range and did not impair bacterial growth in the absence of antibiotic pressure, suggesting a favorable selectivity window. Nonetheless, clinical safety requires rigorous evaluation, particularly potential effects on the eukaryotic homolog Rad51 and DNA-repair processes [32]. Although speculative, in perinatal care—where certain antibiotics (e.g., aminoglycosides, tetracyclines) pose toxicity risks—low-dose BR co-treatment with β-lactams might help delay resistance without increasing fetal toxicity; in scenarios requiring polymyxins, BR could extend treatment windows by delaying resistance resurgence [29]. Additional regulatory layers such as codon-usage bias may also affect RecA translation under stress and warrant investigation with codon-optimized constructs [33,34], alongside in vivo validation.

In summary, our data indicate that targeting RecA with BR attenuates early-stage spontaneous resistance across multiple antibiotic classes by constraining recombination-linked tRNA upregulation and curbing translational/energetic capacity. This study offers a practical framework—phenotype, transcriptome, protein, and tRNA metrics across multiple antibiotics—for evaluating resistance-suppressing adjuvants. Future work should refine dosing strategies, explore co-targets that limit persistence, and pursue in vivo efficacy to translate this approach into clinical antimicrobial regimens.

## 5. Patents

This work has resulted in the following patent application:

Zhang, G.; Ma, J.; Yu, Z. A method for inhibiting spontaneous development of drug resistance in bacteria. CN118846076A, China. Published on 29 October 2024. Patent pending [35].

## Data Availability

The transcriptome data supporting this study are available in the NCBI Sequence Read Archive (SRA) under project accession number PRJNA1200949. The data can be accessed using the following reviewer link: https://www.ncbi.nlm.nih.gov/bioproject/PRJNA1200949 (accessed on 3 September 2025).

## Abbreviations

The following abbreviations are used in this manuscript:

BR	BRITE-338733
CIP	Ciprofloxacin
AMP	Ampicillin
TET	Tetracycline
PMB	Polymyxin B
KAN	Kanamycin
AR	antimicrobial resistance
MDR	multidrug-resistant
XDR	extensively drug-resistant
MIC	minimum inhibitory concentration
ANOVA	One-way analysis of variance
KW	Kruskal–Wallis
GO	Gene ontology
RPKM	reads per kilobase per million mapped reads
DEGs	differentially expressed genes
BP	biological process
MF	molecular function
CC	cellular component

## References

[1] Davies J., Davies D. (2010). Origins and Evolution of Antibiotic Resistance. Microbiol. Mol. Biol. Rev..

[2] Naghavi M., Vollset S.E., Ikuta K.S., Swetschinski L.R., Gray A.P., Wool E.E., Aguilar G.R., Mestrovic T., Smith G., Han C. (2024). Global burden of bacterial antimicrobial resistance 1990–2021: A systematic analysis with forecasts to 2050. Lancet.

[3] Karnwal A., Jassim A.Y., Mohammed A.A., Al-Tawaha A.R.M.S., Selvaraj M., Malik T. (2025). Addressing the global challenge of bacterial drug resistance: Insights, strategies, and future directions. Front. Microbiol..

[4] Centers for Disease Control and Prevention (2013). Antibiotic Resistance Threats in the United States.

[5] Hooper D.C. (2001). Emerging mechanisms of fluoroquinolone resistance. Emerg. Infect. Dis..

[6] Luria S.E., Delbrück M. (1943). Mutations of Bacteria from Virus Sensitivity to Virus Resistance. Genetics.

[7] Nikaido H. (2009). Multidrug resistance in bacteria. Annu. Rev. Biochem..

[8] Fang H., Zeng G., Zhao J., Zheng T., Xu L., Gu W., Liu Y., Zhang J., Sun X., Zhang G. (2021). Genome recombination-mediated tRNA up-regulation conducts general antibiotic resistance of bacteria at early stage. Front. Microbiol..

[9] Zhong J., Xiao C., Gu W., Du G., Sun X., He Q.Y., Zhang G. (2015). Transfer RNAs Mediate the Rapid Adaptation of *Escherichia coli* to Oxidative Stress. PLoS Genet..

[10] Bell J.C., Kowalczykowski S.C. (2016). RecA: Regulation and Mechanism of a Molecular Search Engine: (Trends in Biochemical Sciences, June 2016, Vol. 41, No. 6, 491–507). Trends Biochem. Sci..

[11] Diaz-Diaz S., Yerbes P., Recacha E., de Gregorio-Iaria B., Pulido M.R., Romero-Muñoz M., Docobo-Pérez F., Pascual A., Rodríguez-Martínez J.M. (2023). RecA inactivation as a strategy to reverse the heteroresistance phenomenon in clinical isolates of *Escherichia coli*. Int. J. Antimicrob. Agents.

[12] Thi T.D., López E., Rodríguez-Rojas A., Rodríguez-Beltrán J., Couce A., Guelfo J.R., Castañeda-García A., Blázquez J. (2011). Effect of *recA* inactivation on mutagenesis of *Escherichia coli* exposed to sublethal concentrations of antimicrobials. J. Antimicrob. Chemother..

[13] Zabłotni A., Schmidt M., Siwińska M. (2024). The SOS Response Activation and the Risk of Antibiotic Resistance Enhancement in Proteus spp. Strains Exposed to Subinhibitory Concentrations of Ciprofloxacin. Int. J. Mol. Sci..

[14] Huerta-Uribe A., Marjenberg Z.R., Yamaguchi N., Fitzgerald S., Connolly J.P.R., Carpena N., Uvell H., Douce G., Elofsson M., Byron O. (2016). Identification and Characterization of Novel Compounds Blocking Shiga Toxin Expression in *Escherichia coli* O157:H7. Front. Microbiol..

[15] Sexton J.Z., Wigle T.J., He Q., Hughes M.A., Smith G.R., Singleton S.F., Williams A.L., Yeh L.A. (2010). Novel Inhibitors of *E. coli* RecA ATPase Activity. Curr. Chem. Genom..

[16] Ma J., Shang K., Xu L., He Q.Y., Zhang G. RecA Inhibitor Mitigates Bacterial Antibiotic Resistance. Proceedings of the 3rd International Electronic Conference on Microbiology (ECM 2025).

[17] (2025). E. coli Genetic Resources at Yale CGSC.

[18] American Type Culture Collection (ATCC) (2025). https://www.atcc.org.

[19] Watanakunakorn C. (1988). In-vitro induction of resistance in coagulase-negative staphylococci to vancomycin and teicoplanin. J. Antimicrob. Chemother..

[20] Andrews J.M. (2001). Determination of minimum inhibitory concentrations. J. Antimicrob. Chemother..

[21] Zhang G., Zhang Y., Jin J. (2021). The Ultrafast and Accurate Mapping Algorithm FANSe3: Mapping a Human Whole-Genome Sequencing Dataset Within 30 Minutes. Phenomics.

[22] Kolde R. pheatmap: Pretty Heatmaps. R Package Version 1.0.12. https://CRAN.R-project.org/package=pheatmap.

[23] Makowski D., Ben-Shachar M., Lüdecke D. (2019). bayestestR: Describing Effects and their Uncertainty, Existence and Significance within the Bayesian Framework. J. Open Source Softw..

[24] Love M.I., Huber W., Anders S. (2014). Moderated estimation of fold change and dispersion for RNA-seq data with DESeq2. Genome Biol..

[25] Yu G., Wang L.G., Han Y., He Q.Y. (2012). clusterProfiler: An R package for comparing biological themes among gene clusters. Omics J. Integr. Biol..

[26] Uttatree S., Charoenpanich J. (2016). Isolation and characterization of a broad pH- and temperature-active, solvent and surfactant stable protease from a new strain of Bacillus subtilis. Biocatal. Agric. Biotechnol..

[27] Wigle T.J., Sexton J.Z., Gromova A.V., Hadimani M.B., Hughes M.A., Smith G.R., Yeh L.A., Singleton S.F. (2009). Inhibitors of RecA activity discovered by high-throughput screening: Cell-permeable small molecules attenuate the SOS response in *Escherichia coli*. J. Biomol. Screen..

[28] Fuss J.O., Tsai C.L., Ishida J.P., Tainer J.A. (2015). Emerging critical roles of Fe–S clusters in DNA replication and repair. Biochim. Biophys. Acta (BBA)-Mol. Cell Res..

[29] Cronan J.E. (2018). Advances in synthesis of biotin and assembly of lipoic acid. Curr. Opin. Chem. Biol..

[30] Jozefczuk S., Klie S., Catchpole G., Szymanski J., Cuadros-Inostroza A., Steinhauser D., Selbig J., Willmitzer L. (2010). Metabolomic and transcriptomic stress response of *Escherichia coli*. Mol. Syst. Biol..

[31] Liao H., Yan X., Wang C., Huang C., Zhang W., Xiao L., Jiang J., Bao Y., Huang T., Zhang H. (2024). Cyclic di-GMP as an antitoxin regulates bacterial genome stability and antibiotic persistence in biofilms. eLife.

[32] Lin Z., Kong H., Nei M., Ma H. (2006). Origins and evolution of the *recA*/RAD51 gene family: Evidence for ancient gene duplication and endosymbiotic gene transfer. Proc. Natl. Acad. Sci. USA.

[33] Quax T.E.F., Claassens N.J., Söll D., van der Oost J. (2015). Codon Bias as a Means to Fine-Tune Gene Expression. Mol. Cell.

[34] Plotkin J.B., Kudla G. (2011). Synonymous but not the same: The causes and consequences of codon bias. Nat. Rev. Genet..

[35] Zhang G., Ma J., Yu Z. (2024). A Method for Inhibiting Spontaneous Development of Drug Resistance in Bacteria.

